# Acceptability and Utility of a Web-Based Patient-Completed Clinical Decision Aid for the Differential Diagnosis of Transient Loss of Consciousness: Qualitative Interview Study

**DOI:** 10.2196/67608

**Published:** 2025-07-24

**Authors:** Alistair Wardrope, Lindsay Blank, Melloney Ferrar, Steve Goodacre, Daniel Habershon, Markus Reuber

**Affiliations:** 1 Division of Neuroscience, University of Sheffield, Royal Hallamshire Hospital, Glossop Road, Sheffield, S10 2JF, United Kingdom, 44 01142434343 ext 12118; 2 Sheffield Teaching Hospitals NHS Foundation Trust, Sheffield, United Kingdom; 3 Sheffield Centre for Health and Related Research (ScHARR), University of Sheffield, Sheffield, United Kingdom

**Keywords:** user experience, virtual medicine, triage, acceptability, qualitative research, qualitative interview, qualitative interview study, online patient-completed clinical decision aids, online, clinical decision, patient risk, burden, consciousness, interview, thematic analysis, usability, clinical decision-making, virtual, medicine, digital, technology, digital health

## Abstract

**Background:**

Web-based patient-completed clinical decision aids (CDAs) have the potential to reduce inefficient resource use and patient risk in acute and emergency settings while minimizing additional clinician time burdens. However, such interventions must be acceptable for use by their target audience—patients.

**Objective:**

The objective of this study is to assess acceptability and utility to patients of a novel online patient-completed CDA for the differential diagnosis of transient loss of consciousness (TLoC).

**Methods:**

Within a larger validation study of a patient-completed CDA, we conducted nested qualitative semistructured interviews with a purposive sample of 20 patients who used the CDA in the study and performed thematic analysis of interview transcripts.

**Results:**

We identified 11 themes within the data: 3 addressing the content of the CDA, 3 addressing the online implementation, and 4 addressing usability and acceptability of the CDA. Respondents generally felt an online CDA was easy to complete and acceptable, though they felt that increased options to personalize descriptions of their experience would be helpful and offered guidance on how to make it a more useful resource for patients as well as clinicians. We present good practice points for the design of patient-completed online CDAs on the basis of our thematic analysis.

**Conclusions:**

Findings suggest that patient-completed CDAs may be accessible and feasible in acute and emergency settings, though further research is needed to explore their real-world usability. In designing such tools, clinicians should endeavor to maintain their accessibility for all relevant patient groups and to use them to provide direct patient benefit, as well as to support clinical decision-making, for example, through simultaneous patient-directed outputs.

## Introduction

With rising demand for health care services and increasing resource pressures, new tools are needed to ensure more efficient and effective care delivery. Clinical decision aids (CDAs) are sets of instructions that use accessible clinical data to provide clear guidance on appropriate next steps in patient management. Appropriately applied, they reduce inefficient resource use and patient risk in acute and emergency settings [1].

One barrier to the application of CDAs is clinician time burden. This could be addressed through patient self-completion prior to or following clinician assessment. Existing work demonstrates the feasibility of inviting patients to participate in the application of CDAs for their own care [2,3]. Increasingly mobile health technologies such as tablet or smartphone applications are allowing for patient-led data collection and CDA deployment [4]. These have largely been used in outpatient and elective settings for the care of patients with chronic health problems [4-6], but there is evidence that such tools are feasible in the Emergency Department (ED) setting [7-9] and no less acceptable than clinician-completed use of the same tool [10]. Given that around 9 in 10 patients attending EDs in England in 2018‐19 spent over an hour in the department [11], ED attendances provide ample opportunity for self-administration. Such CDAs could be completed in the waiting room prior to assessment, using departmental tablets (as in this study), or patients’ own smartphones.

However, in order for such complex interventions to become applicable to general clinical practice, it is important to understand acceptability to their target audience—patients. Guidelines on developing such interventions highlight the importance of acceptability assessment as part of the process [12,13].

In this paper, we report the acceptability of a novel patient-completed self-assessment CDA to support differential diagnosis of transient loss of consciousness (TLoC) at first presentation—the Paroxysmal Event Symptoms Questionnaire (PESQ). TLoC is a common emergency presentation, but 20%‐30% are not accurately diagnosed or treated at presentation [14-16]. This has significant resource implications—in 2002, the annual direct medical costs of epilepsy misdiagnosis in England and Wales were estimated to be £29,000,000 (US $40-46 million in 2002) [17].

However, the use of CDAs in the diagnosis and management of TLoC presentations has thus far been limited. There are no well-supported criteria for the distinction between all common causes of TLoC. While there are some candidate CDAs designed to discriminate between syncope and bilateral tonic-clonic seizures [18,19] or epilepsy and functional/dissociative seizures [20,21], none covers all relevant presentations, nor has been prospectively validated in mixed TLoC populations. This lack of robust criteria is acknowledged in the UK National Institute of Clinical Excellence guidance on TLoC assessment, which provides very limited suggestions of features that should lead doctors to “suspect epileptic seizures” based on low- and very low-quality evidence and expert opinion alone [22].

In this paper, we use patient experiences of interacting with the PESQ to elucidate understanding of patient requirements of an online, patient-completed CDA. We performed a concurrent nested qualitative study within a larger quantitative research project developing and validating the PESQ in a first-presentation TLoC population. We aimed to identify whether users found the tool acceptable for use, easy to use, and relevant to their clinical presentation.

## Methods

The data reported in this paper come from interviews conducted as the qualitative arm of a mixed methods study developing and validating the PESQ for use in a first-presentation TLoC population [23]. The design, setting, participants, interviews, and analysis are described fully elsewhere [24].

### Setting and Participants

We conducted the study within a single large hospital Trust in the United Kingdom, screening all patients presenting to the ED with TLoC or referred to first seizure or syncope clinics within the window of February 10, 2022, to January 9, 2023. All participants recruited to the quantitative study were also invited to interview; we approached those who consented to interview sequentially.

We had a provisional target of 30 participants, using empirical data showing 24 participants reliably achieves saturation, the narrow specification of subject matter, and the study team’s expertise in the subject matter [25].

### Instruments

All participants in the quantitative study completed the PESQ at or shortly after presentation to the ED or primary care with TLoC. The majority completed it using a simple online platform. The PESQ comprises a brief (52-item) questionnaire comprising 4 demographic questions, 13 regarding patient medical history, and 35 peri-ictal symptoms. The online platform delivered these with sequential presentation of each question, with the option for binary yes or no responses (except for the demographic questions of age and years of education, which allowed for any integer answer). Participants could skip questions or navigate back and forth between them. Multimedia Appendix 1 lists PESQ items. For this study, all PESQ data were stored in anonymized fashion on a university secure database.

Patients could complete the questionnaire at the time of assessment and presentation using a tablet computer provided by the study team or else at home after their initial presentation through a simple browser-based application.

### Interviews

Two researchers (AW and DH) conducted remote (video, via Microsoft Teams, or telephone) semistructured interviews following a predefined interview schedule (Multimedia Appendix 2). Interviews lasted between 30 minutes and 1 hour. Interviewers noted initial reflections in contemporaneous logs, to support reflexive engagement with later analysis. An independent, nonclinical, professional transcription service transcribed all interviews for subsequent analysis.

### Analysis

We undertook thematic analysis [26] of transcribed interviews [27]. One researcher (LB; an experienced qualitative researcher with no clinical background or experience of TLoC) imported transcribed interviews into NVivo (Lumivero), and 2 researchers (LB and AW; a specialty registrar [senior resident] in neurology, with prior experience as a core trainee [junior resident] in cardiology and emergency medicine) developed and refined themes through iterative coding.

We assessed for saturation through interim analyses, stopping recruitment when saturation was reached.

### Ethical Considerations

We preregistered the study protocol on ClinicalTrials.gov (NCT05367999). Ethical approval came from the NHS Health Research Authority Edgbaston Research Ethics Committee (IRAS: 304114). We report results in line with the SRQR (Standards for Reporting Qualitative Research; checklist provided in Checklist 1) [28].

## Results

### Participants and Demographics

Of 2811 potential participants screened for recruitment, 1181 were eligible. Of these, 186 responded to the invitation to participate, and 133 also consented to approach for an interview. We approached 40 participants for interviews, aiming for diversity of age and gender. After 20 interviews, we achieved data saturation.

Of interview participants, 14 (70%) received final diagnoses of syncope, and 6 (30%) epilepsy. In total, 12 participants (60%) were female. The median age was 69 (range 17-90) years. Interviews were held a median of 69 (range 35‐283) days from initial presentation. Table 1 displays full participant demographics.

**Table 1.** Demographics and diagnoses of interview participants. Participants were recruited from all adult referrals to the Emergency Department, Acute Medical Unit, and syncope or seizure clinics with a first transient loss of consciousness (TLoC). They participated in semistructured interviews regarding their experiences of interacting with a prototype patient-completed clinical decision aid for the differential diagnosis of TLoC.

ID	Age	Gender	Diagnosis
TL095	27	F	Syncope
TL099	69	F	Syncope
TL101	70	M	Syncope
TL106	75	M	Syncope
TL109	90	F	Syncope
TL122	17	F	Epilepsy
TL133	73	M	Syncope
TL146	69	F	Syncope
TL150	78	F	Syncope
TL152	40	M	Syncope
TL157	46	M	Epilepsy
TL169	27	F	Epilepsy
TL173	74	F	Syncope
TL174	24	F	Epilepsy
TL176	36	F	Epilepsy
TL178	82	M	Epilepsy
TL180	79	F	Syncope
TL181	70	F	Syncope
TL184	50	M	Syncope
TL188	44	M	Syncope

### Themes

The semistructured interview protocol included assessment of both participants’ impressions of using the PESQ and of their assessments of its utility and acceptability in clinical practice. We identified 6 themes addressing the former—3 concerning the content of the questions and 3 the design of the tool—and 4 themes addressing the latter. These are summarized in Multimedia Appendix 3.

#### Content of the Questionnaire

##### Appropriate Questions

Respondents were largely happy with the content and clarity of the questions asked and did not suggest significant changes. Several respondents (eg, TL109; TL169) reported that the very act of being asked questions—and given the words with which to frame their experience—helped to remind them of the symptoms they had experienced and find the means with which to express them.

##### Limiting Structure

Other respondents reported that the question structure—in particular, comprising binary yes or no questions—was limiting; they would value the opportunity to elaborate on some of their answers or provide more than yes or no responses. One felt that the questions were too simple and that the opportunity to gain better understanding or “go deeply” into their condition had been missed. More commonly, respondents found they were not always able to give a clear “yes” or “no” answer to some questions; “I appreciate sometimes you wanna force people to make a yes or a no but I didn’t […] find that particularly helpful cos it wasn’t really yes or no answers” (TL101).

##### Questions Not Matching Experience

Some participants queried the content of the questions. These issues came in 3 broad forms: an assumption of multiple blackouts when a person had only experienced one, including too many questions that bore little apparent relevance to their experience; or not including questions that were relevant to their experience.

The PESQ used in this study was derived from a questionnaire originally issued to people with long-standing disorders causing TLoC; as a consequence, many questions referred to “my attacks.” Respondents who had only experienced a single episode found this phrasing confusing.

Others (eg, TL184) noted that many of the questions referenced experiences or historical characteristics that did not appear relevant to them and found the process of going through lots of questions to give them negative responses difficult; it was “just a little bit frustrating to go through and go no I’ve not had that and, no I’ve not seen that, no this hasn’t happened to me.” The face validity of the questionnaire to respondents appeared to fall when this was the case.

Lastly, some felt that the questions did not sufficiently cover the aspects of their experience—or their general background—that they felt most important. This might be individual symptoms (“I’d lost control of my bladder [but] there was nothing like that to say yes or no” [TL173]), comorbidities, eg, coeliac disease (TL146), or the social milieu of their TLoC (“the first one was actually different to the second one […] if my wife hadn’t […] kinda like pushed me and shouted at me, I would have probably gone out” [TL101]).

### Design of the Tool

#### Ease of Use

In nearly all cases, respondents reported that the tool was easy to use and the interface easy to operate. A number of respondents (eg, TL150) noted that the interface was accessible for people who were “not a hundred percent au fait with the […] computer” (ie, low self-evaluated digital literacy). There were no technical issues raised with the exception of 1 respondent (TL173) who had trouble with an outdated internet browser rather than with the tool itself. The simple format with few options and little text per page was highlighted as making navigation straightforward.

#### Language Used

Respondents overwhelmingly felt that the questions they were answering made sense to them. Some struggled with individual items. This arose in some cases due to language use—either specific terms (eg, “febrile seizures” for TL101) or, more generally, language of a higher complexity that was not “plain English” (TL178).

#### Going Beyond the Questionnaire

Several participants made suggestions regarding how the tool could be extended beyond the original questionnaire and its intended applications. For some, this concerned other means in which users could narrate their experience than just the questionnaire, for example, supplementing the yes or no questions with free-text descriptions of ictal symptoms. Others felt that the tool could be adapted to support users’ informational needs. This could be as simple as providing users with a record of their answers for a reference description of ictal experience they could turn to later (TL099); others thought that after questionnaire completion would be a helpful time for users to be provided with further information about TLoC—either the model’s predicted likely diagnosis for the user, with tailored information depending on cause (TL106), or general information regarding the TLoC assessment pathway; users wanted support in “explaining […] the process […] what the next steps would be […] cos I don’t want to have to keep going to my GP or the A+E every time I experience these episodes” (TL169).

### Clinical Utility and Acceptability

#### Expectation of Benefit

Many respondents did think that the tool would be helpful in an emergency setting. Those still in situations of diagnostic uncertainty (eg, TL173) felt that it might streamline the assessment pathway or give them answers sooner. Some (eg, TL095) saw the tool as licensing access to definitive investigations. Others (eg, TL169) thought the existence of the questionnaire itself—independent of any diagnostic predictions it could generate—would improve the emergency assessment, by overcoming challenges related to the description of episodes—“it’s hard to put things into words yourself…there can be…a lot of confusion at appointments, trying to explain the…episodes.”

#### Unable to Gauge Benefit

Others felt they were unable to assess the potential for benefit in the emergency setting. For some (eg, TL099) this related to the user-generated nature of the questionnaire outputs, which they saw as “my interpretation,” contrasted with the “objective method” of medical assessment (TL099). For others, the difficulty rather came from the confusion or disorientation they experienced at the time of assessment, making this a difficult issue for them to adjudicate.

Many patients reported feeling confused or disoriented when asked to complete the questionnaire, even though this was done subsequently in their own home. In at least one case the respondent reported that they had difficulty remembering actually doing it. This in itself raises questions about whether the participants would have been able to complete the questionnaire in an ED shortly after their blackout occurred and whether they would have required help from someone who had witnessed their blackout in order to answer the questions accurately.

#### Unlikely to Benefit

Two respondents did not think there was any real prospect for the tool improving patient care; however, in both cases, this was because they were entirely satisfied with the standard of care and felt nothing needed to be added to their assessment.

#### A Tool to Benefit Clinicians and Researchers, Not Patients

Two respondents challenged the question of benefit by drawing a distinction between clinician (or researcher) benefit, on the one hand, and patient benefit, on the other. For these respondents, the questionnaire treated users as “sources of information” [29]—passive objects to be “read” for the clinician’s or researcher’s epistemic gain—rather than “informants”—peers in the creation and exchange of knowledge. These respondents (eg, TL106) felt that they would want to be able to use the outputs themselves—rather than them simply being passed to clinicians—to experience direct benefit. Information on the assessment pathway was again suggested as one means of realizing patient benefit: “to actually be able to say ‘Look, you know, we’re gonna investigate this and it’s looking like you might have something like this going on so here’s some reading’” (TL184).

## Discussion

### Overview

The experiences of our respondents provide general guidance for the development of patient-completed CDAs in the emergency setting, as well as for subsequent refinement of our specific tool. In general, respondents found a simple online questionnaire tool acceptable and understandable, though they were divided on their assessment of likely clinical benefit. They highlighted some specific features of such tools that they found of particular value; other uses to which such a tool could be put to support the patient navigation of their emergency assessment pathway; and identified important considerations in their design to maximize acceptability and potential benefit.

### Value and Limitations of Structured History-Taking

Some respondents found that having a questionnaire as a prompt for ictal recall and description was itself of value. It could serve as an aide-memoire for particular ictal experiences (“it…highlighted…some of the things that applied to me”; TL109) or even could provide the conceptual resources for articulating experiences that are “hard…to put into words yourself, not understanding it” (TL169). Previous work comparing structured and open interviews to extract seizure histories has demonstrated the value of such systematic closed questioning to enhance the yield of ictal history-taking [30,31] and support patients’ self-understanding and interpretation of experience [32]; our respondents suggest similar benefits can be found when answers are elicited in a patient-administered, self-report format (more practical in resource-limited settings, with online capture easily facilitating computer-aided interpretation of results). This is consonant with previous work demonstrating that, in general, electronic data collection is more effective than paper-based administration of the same questionnaires [6,33].

However, our participants also highlighted the limitations of this format. They described various ways in which a finite symptom list, with binary yes or no answers, limited their ability to articulate their experience—whether from a surfeit of irrelevant questions, a lack of relevant ones, or feeling constrained by the insistence on the dichotomous presence or absence of a given experience, regarding which they might feel ambivalent or uncertain. Part of this issue may stem from a failure of the study team to articulate fully the purpose of the questionnaire—not to capture a complete description of the person’s TLoC experience, but rather to use certain previously identified highly discriminating features to predict likely etiology of the TLoC—but in part their concerns reflect those found more widely in the experiences of those completing symptom questionnaires. Exploring conditions as diverse as breathlessness and depression, patients completing symptom questionnaires describe ambivalent reactions to the depictions of their experience thus captured [34,35]. They may seek to reformulate questions, recontextualize them (as with TL101’s avoiding of the present or absent dichotomy, or TL106’s looking for follow-up exploration of symptoms), or reject their framing (as with TL184 concluding that the research was not directed toward their presentation) [35].

Beyond just highlighting this issue, our data suggest means to address it. Respondents were clear on the need for such a CDA to complement, not replace, in-person clinical assessment; and they suggested that a more nuanced description of their experience could be captured by complementing the structured questions with free-text symptom reporting. The combination of questionnaires and AI-supported analysis of free speech seizure descriptions may address this potential deficiency of an approach based on closed questions alone [36,37].

### Emergency Department Implementation of Patient-Completed CDAs

While much of the research on patient-completed CDAs has focused on their role in the management of chronic conditions [4,38], there is increasing recognition of the opportunities they offer in acute and emergency care. In particular, several studies have explored the role of patient-completed symptom checkers in supporting triage at the ED front door [8,9,39]. While self-triage CDAs for unselected presentations have been found to perform inadequately [39], quantitative assessments of acceptability and utility in this setting have generally shown patients are willing and able to interact with such tools [8,9]. Our study enriches this finding with qualitative data exploring patient preferences for interacting with such tools. As well as having a simple, navigable interface, they reported a preference for patient-facing outcomes to demonstrate benefit to them (explored more below).

In order to develop CDAs fit for supporting emergency and primary care practice, it is important to understand not just how the CDA performs in ideal conditions, but also how it will perform in the “real world” and how clinicians will use it in practice [40,41]; in particular, what are the barriers to and facilitators of physician uptake of a new CDA. Not all factors affecting uptake of CDAs depend on the design of the CDA itself; from a thematic analysis of semistructured interviews with emergency clinicians in the UK, Hayes identified that, along with the design of the CDA itself, factors concerning the care providers, the environment of their practice, and the institutions within which they worked might enable or impede widespread use of CDAs [42]. The Ottawa Acceptability of Decision Rules Instrument [43], a validated scale for measuring the likely clinical acceptability of new candidate CDAs for emergency care use, similarly considers factors both related to the CDA itself and the context in which it is to be used. The tool-related factors that affect uptake of CDAs can broadly be divided into 3 main areas of concern: how useful the CDA is; how accessible it is to apply in the working context; and how justifiable its decisions are—whether to patients, other clinicians, or in legal contexts. The 5 core principles of CDA design dictate that they must provide the right information to the right person in the right format through the right channel at the right time [44,45]. The majority of CDAs are designed to be completed and used by clinicians, but attending to the core principles of CDA design shows that the right information, right person, right format, and right time for information provision could also come through patients. Patient completion of CDAs prior to clinician assessment could empower patients to participate in their care and support shared decision-making [44], while reducing the time burden on clinicians and so improving the utility and accessibility of the CDA. Computerized CDAs already exist for the management of chronic conditions such as hypertension and multiple sclerosis [46,47]; patient-facing checklists have also been used successfully in ED settings to support patient-centered care [2]. Before real-world implementation, the PESQ would require further research addressing these implementation challenges, as well as multicenter validation of its performance.

It is important that such tools be able to be implemented in the ED setting, not only to allow swift and accurate triage but also because it maximizes capture of reliable information. The richness of subjective symptom descriptions decreases with time from the event for many TLoC presentations, reducing the utility of information captured [48]. Our respondents demonstrated this, with several unable to recall elements of their TLoC they reported in the CDA or even using the CDA itself. Allowing patients to return to their answers to support recall for other clinical purposes would be one means of enhancing the patient utility of such tools. Allowing delayed access to review answers through an online platform would also provide patients with the opportunity to reflect on their responses, an option valued in other work on patient-completed CDAs [38].

Implementation in an ED setting would therefore benefit both from an online application to which users could return after their initial attendance and from hardware available in the ED so that patients could interact with the CDA as early as possible in their pathway. In this study we achieved this through making available to participants in the acute hospital setting a tablet PC, through which they could complete the CDA in its browser-based form; but making it online in this fashion also allowed home completion at a later date. However, further implementation research would be necessary to see how successfully this approach could be used for all patients, including, for example, those with language barriers or who were transiently disoriented at the time of presentation.

### Maximizing Patient Benefit From Patient-Completed CDAs

Respondents drew a distinction between clinical benefit—that which achieves the assessing clinician’s aims (in particular, diagnosis)—and patient benefit—addressing the patient’s immediate needs (prominently, information and management of uncertainty) [24]. They proposed ways in which a CDA could be used for patient benefit. Access to answers after the time of completion (eg, through a print-out, or option to log in and return to questionnaire answers) could help them as a reference point in later assessment and care. They also indicated that the time of questionnaire completion would be an ideal opportunity to address patients’ informational needs. An online interface could easily provide patients as well as clinicians information about the results of the CDA and its implications for ongoing management or links to explanations of assessment pathways and interim management while awaiting definitive diagnosis. As reported elsewhere [24], these were predominant concerns of respondents after their first assessment for TLoC, and this represents a significant opportunity to add value to patient-completed CDAs.

While it was not a theme explored extensively by our participants, perhaps because of the study literature clarifying the protocol for research data storage that would differ from widescale health care implementation, data governance will always be important for online CDAs. As with any digital health tool, future iterations of PESQ should consider patient data privacy and security, ensuring compliance with standards such as General Data Protection Regulations (GDPR) or the US Health Insurance Portability and Accountability Act (HIPAA).

Existing tools for supporting CDA development and implementation (such as the Theoretical Domains Framework [38,49] or GUIDES checklist [13]) provide high-level overviews of barriers and enablers of implementation; our data allow for more granular practical guidance. On the basis of our data, we offer a set of good practice points for the development of patient-completed online CDAs, summarized in Table 2.

**Table 2.** Considerations to maximize utility and acceptability of a patient-completed online clinical decision aid (CDA), based on recommendations drawn from thematic analysis of semistructured interviews with adult patients using a prototype CDA at first presentation of transient loss of consciousness.

Theme	Considerations
Content	Language—consider reading age and educational background of your patient group. Pilot questions for intelligibility. Relevance—consider explaining relevance of different items so that patients do not disengage from questions not matching their experience.
Interface	Simplicity—keeping the CDA as simple as possible aids engagement for those not otherwise used to interacting with online or computer interfaces. Flexibility—patients may value the opportunity to adapt their input, eg, through free-text supplements. Compatibility—If patients will complete the CDA on their own device, ensure cross-compatibility with different browsers and both smartphone and desktop access.
Outputs	Data storage—patients may want to be able to retrieve responses and outputs for their own use later. Consider how this can be achieved without increasing time demands or complexity of the interface (eg, by accounts and log-ins). Such storage must be done securely, in line with relevant data protection regulations (eg, GDPR^a^, HIPAA)^b^. Direct patient benefit—patients may have different priorities from clinicians in the use of a CDA. Consider what else might be important to patients using the CDA (eg, information about the clinical problem and self-management or lifestyle advice) and how it can be addressed (eg, with CDA outputs providing links to patient support groups or information and self-management guidance).

a GDPR: General Data Protection Regulations.

b HIPAA: Health Insurance Portability and Accountability Act.

### Limitations

We note some important limitations. The inclusion criteria for the quantitative study necessarily left some groups underrepresented, particularly those with insufficient English language proficiency to complete the questionnaire or learning disabilities that would similarly make self-completion difficult. To an extent this is by design—since cross-language and cross-cultural validation would be necessary anyway after development of the English-language version of such a CDA prior to its employment in different languages. We did include within our sample a diverse age range, including older populations who may be less familiar with online tools—it is a strength of this study to note that even those who self-described as “not a hundred percent au fait” with computer use were able to use the tool without difficulty. Previous research has suggested that older adults are less likely to engage with tablet-based mobile health interventions [4], but that when they do are no less likely to complete them than younger patients, suggesting that perceptions of digital literacy may be lower amongst older patients than actual inability to engage with such tools. To remove such barriers, CDA design can emphasize aspects more likely to be acceptable in this demographic [50], such as simple user interfaces and minimizing numbers of clicks and scrolls, without intrusive features like pop-ups.

We also recruited only a small proportion of those eligible to participate. Anecdotally, the study team noted that recruitment rates appeared higher when potential participants were approached directly and contemporaneously with their assessment, rather than retrospectively by letter. Future research could assess differential recruitment rates and effects on sample representativeness of different recruitment pathways.

Additionally, as a measure of acceptability of such a CDA, we only describe qualitative findings; further work could complement this with quantitative data, both survey responses identifying users’ evaluations of acceptability (along dimensions guided by the data presented in this study) and objective measures, eg, noncompletion of the questionnaire.

### Further Work

In reporting the main quantitative outcomes of this study, we discuss the further validation and implementation research necessary to determine the practical value of the use of such a CDA in routine practice [23]. The results from this study highlight the need to combine this with further research refining the acceptability and utility of the CDA to patients and clinicians. Furthermore, before application in different cultural contexts and different languages, cross-language and cross-cultural validation would be required. Our present research provides guidance—summarized in Table 2—for how such a program of research could proceed.

### Conclusions

In this study we find that an online, patient-completed questionnaire tool to support a CDA for the differential diagnosis of TLoC is generally acceptable to users, though opinions diverge on the likely utility of such a tool. Respondents highlighted the distinction between clinician and patient benefit—noting that while interests overlap, they are not coextensive—and offered means to improve the tool such that, in addition to supporting a CDA for clinical benefit, it could address the informational needs of patients at the time of their first assessment for TLoC.

## Supplementary material

10.2196/67608 Multimedia Appendix 1 Paroxysmal Event Symptoms Questionnaire.

10.2196/67608 Multimedia Appendix 2 Interview schedule.

10.2196/67608 Multimedia Appendix 3 Summary table of themes and sub-themes with illustrative quotations.

10.2196/67608 Checklist 1 SRQR (Standards for Reporting Qualitative Research) checklist.

## Abbreviations

CDA: clinical decision aid
ED: emergency department
GDPR: General Data Protection Regulations
HIPAA: Health Insurance Portability and Accountability Act
PESQ: Paroxysmal Event Symptoms Questionnaire
SRQR: Standards for Reporting Qualitative Research
TOLC: transient loss of consciousness
