# Supplementary material for: Acceptability and Utility of a Web-Based Patient-Completed Clinical Decision Aid for the Differential Diagnosis of Transient Loss of Consciousness: Qualitative Interview Study
Source: JMIR Form Res. 2025 Jul 24;9:e67608. doi: 10.2196/67608 (PMC12289296; doi:10.2196/67608)
Supplement: Multimedia Appendix 1 [file formative-v9-e67608-s001.docx]

Paroxysmal event symptom questionnaire (PESQ) and paroxysmal event witness questionnaire (PEWQ)

Supplementary eAppendix 1

# PESQ

## Personal background

1. What is your age in years?
2. For how many years were you in formal education?
3. How would you describe your gender? (Man/woman/other gender)

## Medical history

Which of the following conditions do you have or have you had?

1. Febrile seizures in childhood
2. Episodes of chest pain or tightness
3. Breathlessness unrelated to exercise
4. Palpitations
5. Brain tumour
6. Head injury with loss of consciousness
7. Lightheaded spells
8. Brief jerks of the arms or legs
9. Poor coordination
10. Chronic fatigue
11. Gastro-oesophageal reflux disease (GORD)
12. Learning disability
13. Family history of seizures
14. Any mental health condition (diagnosed by a healthcare professional)

## Symptoms

Which of the following do you experience in your blackouts?

1. My attacks come on when I am asleep
2. The sight of blood or needles triggers my attacks
3. May attacks are associated with sitting or standing for a long time
4. My attacks are associated with emotional stress
5. My attacks are triggered by sleep deprivation
6. My attacks are triggered by exposure to bright lights
7. My attacks are triggered by frustration
8. My attacks build up gradually
9. In my attacks I seem to be controlled by someone outside me
10. In my attacks I have a sense or feeling as if I have seen something before when I know I have not
11. In my attacks I have a sense of feeling as if I’ve never seen something before when I know I have
12. In my attacks I feel sick
13. I feel hot or cold in my attacks
14. In my attacks I experience tingling or numbness of my skin
15. During my attacks I hear things that are not really there
16. During my attacks I smell things that are not really there
17. In my attacks my mouth goes very dry
18. During my attacks I can see or hear the people around me
19. In my attacks I am conscious but cannot react to things
20. In my attacks I drift in and out of consciousness
21. I am aware of shaking uncontrollably during an attack
22. During my attacks I feel as if I am outside my body
23. My attacks make time go in slow motion
24. In my attacks I feel like I am choking or very short of breath
25. During my attacks I have memories of a past bad experience which I cannot stop
26. During my attacks I am frightened I am going to die
27. My attacks are like a burst of electricity in my brain
28. My attacks are painful like a hammer blow
29. My attacks feel like a knife through the head
30. I wake from my attacks with a cut tongue
31. After my attacks my muscles ache
32. After my attacks I feel very confused
33. I want to know what has happened when I black out
34. After my attacks I feel relieved
35. Afterward I have no idea that I have had an attack

# PEWQ

1. The attacks involve chewing, smacking, or licking movements of the mouth and lips
2. The attacks involve fiddling, picking, or fumbling movements of the hands
3. The attacks involve scratching or bicycling movements of the legs
4. In the attacks the head moves rapidly from side to side
5. The attacks involve violent shaking of the arms and legs
6. The attacks involve movements that are not rhythmic, or wax and wane
7. The attacks involve violent thrusting of the hips
8. During the attack, arms and legs are limp
9. During the attack, arms and legs are rigid
10. During the attack, the person stares to the left or right
11. During the attack, the eyelids flutter
12. Shaking of the arms and legs goes on for over 1 minute
13. The attacks involve movement into unusual positions
14. The skin or lips looked pale during the attack
15. The attack started with an unusual scream or cry
16. Breathing stopped during the attack
17. Breathing was shallow or quiet after the attack
18. Breathing was like snoring after the attack
