# Supplementary material for: Acceptability and Utility of a Web-Based Patient-Completed Clinical Decision Aid for the Differential Diagnosis of Transient Loss of Consciousness: Qualitative Interview Study
Source: JMIR Form Res. 2025 Jul 24;9:e67608. doi: 10.2196/67608 (PMC12289296; doi:10.2196/67608)
Supplement: Multimedia Appendix 3 [file formative-v9-e67608-s003.docx]

Multimedia Appendix 2

Tabular summary of themes and illustrative quotations.

| **TOPIC** | **THEME** | **ILLUSTRATIVE QUOTATIONS** |
| --- | --- | --- |
| 1. **Content of questionnaire** | - 1. Appropriate questions | “*It was relevant and applicable.”* [TL095]  *“I’m not medical myself so I, I wouldn’t know all the ins and outs but you could see that it was, there were some that were sort of leaning towards is it a heart issue or is it an epilepsy issue or is there, you know, other aspects of, of medical stuff going on. So yeah, you could see what it was aiming for.”* [TL184]  A: *“Well it just highlighted, you know, some of the things that applied to me.”* [TL109] |
|  | - 1. Limiting structure | “*I found it very narrow being yes and no […] I appreciate sometimes you wanna force people to make a yes or a no but […] it wasn’t really yes or no answers.”* [TL101]  “*[A]t the end of a lot of questionnaires it’s one, two, three and at the bottom ‘other’ if there’d have been an ‘other’ possibly, you know, a blank one, if there’s anything that we’ve not covered could you please fill in sorta thing, if you get me drift?”* [TL146]  “*Generally yes; in fact probably the only feeling I’ve got about it was it was perhaps a little bit too simple and didn’t go into things too deeply.”* [TL106] |
|  | - 1. Questions not matching experience | “*I mean they did, they were sort of appropriate but not, they seemed to be sorta more […] generalised than […] my specific case.”* [TL106]  *“[I]t was just a little bit frustrating to go through and go no I’ve not had that and, no I’ve not seen that, no this hasn’t happened to me, and it seemed to be lots of nos [...] Iwas more sort of thinking […] something’s happened to me and there’s obviously some research going on around this […], and what’s happened to me doesn’t seem to fit with what the research is looking at.”* [TL184]  *“Obviously it’s a generic one and […] there are people that will have had other issues […] I’m not saying that there’s anything wrong with it but it […] wasn’t relevant to my experience”.* [TL099] |
| 1. **Design of tool** | - 1. Ease of use | *“If I used it it must have been OK […] because I’m not a hundred percent au fait with the […] computer.”* [TL150]  *“I think the content on the website is very, is very clear […] easy to read, and the questions are worded very well…… I think it was like very easy to like navigate.”* [TL169]  “*It was* [‘a battle’ to get on] *and then, as I say, the second time I tried to get on for my son to do it we just couldn’t manage it.”* [TL173] |
|  | - 1. Language used | *“You know, the way they were worded wasn’t [...] plain English […] wording was, you know, hospital speech, I would say […] If it had been in plain English it’d have been easier to understand”.* [TL178]  *“I mean even though I come from [a healthcare background], I don’t know what febrile seizures means […] for instance number five, my attacks are triggered by sleep deprivation; well it might have been better to have said associated cos […] it depends what you’re wanting to know, because I’d not been sleeping well for [...] quite a long time prior to these attacks and so I wouldn’t say it’s triggered, […] but it could be associated with it.”* [TL101] |
|  | - 1. Going beyond the questionnaire | *“[I]f I’d been sent a questionnaire in the post I would have probably have done it and posted it back and kept a record of it for myself as well. I, if I’ve done it online does that mean there is a record of it for me somewhere?”* [TL099]  *“[I]f I could get some further information on what was causing the blackouts or likely to in my particular circumstances, yeah, I, I think that’s got to be a benefit.”* [TL106]  *“I think as well maybe in […] the questionnaire kind of explaining […] the process maybe or like what the next steps would be, cos I think waiting in this kinda period of not knowing what it […] is and not knowing when you’re kind of gonna be seen it […] makes it […] a little bit difficult cos obviously I don’t want to have to keep going to my GP or the A & E every time I experience these episodes.”* [TL169] |
| 1. **Clinical utility** | - 1. Expectation of benefit | “*I think definitely one hundred percent, because it’s hard to put things into words yourself, not understanding it; to be able to kind of have those questions can make you kind of look back and provide the answers for that question, whereas when I went to the GP it was so difficult to kind of explain exactly what it was.*” [TL169]  *“Probably, because it might have then led to, more questions might have been asked which might have led to more investigations.”* [TL095] |
|  | - 1. Unable to gauge benefit | *“I don’t know […] I suppose this is my […] interpretation and A & E is an objective method, isn’t it, of seeing what I, how I am.”* [TL099]  *“To be honest, I can’t remember, I can’t remember doing it. I just remember, I remember doing the questionnaire but I don’t remember getting out of it really.”* [TL099]  “*I looked at it when you sent it through and I was thinking I don’t really remember filling it out. I, I remember thinking I need to fill that out, but it, certainly in the week or two after the seizure lots of things were just coming and going.*” [TL157] |
|  | - 1. Unlikely to benefit | “*“I, I don’t think so. I think that on admission it was, you know, I was dealt with very, very well and everything they did was very thorough and the explanations they gave were, were really clear; so I, I knew what was going on and I knew that the right things were being investigated.”* [TL184]  A: “*No, I don’t think so.”*  Q: “*OK, that’s helpful to know. So you think, don’t think it would have added much to how things went and it would have gone more or less the same anyway?”*  A: “*I think so, yeah.”* [TL181] |
|  | - 1. A tool for clinicians and researchers, not patients | “[I]*t’s more sort of, if you like, helpful to you and your colleagues rather than myself doing the questionnaire because obviously I’m inputting the information to you rather than using the information meself, if you know what I’m saying?”* [TL106]  *“[I]f I’d come in with, you know, a classic heart issue or a classic epilepsy type issue or, you know, some of the other things that you might be able to almost screen for with a questionnaire, to actually be able to say “Look, you know, we’re gonna investigate this and it’s looking like you might have something like this going on so here’s some reading” and, and to be able to give that sort of pre-warning almost to the medical team that it’s looking like this is an issue, then I think that would be really helpful. It’s just in, just in my case it, it probably wouldn’t match whatever you had, so…”* [TL184] |
